# Genome-wide characterization of the SPL gene family involved in the age development of *Jatropha curcas*

**DOI:** 10.1186/s12864-020-06776-8

**Published:** 2020-05-20

**Authors:** Niu Yu, Jin-Chang Yang, Guang-Tian Yin, Rong-Sheng Li, Wen-Tao Zou

**Affiliations:** Key Laboratory of State Forestry Administration on Tropical Forestry Research, Research Institute of Tropical Forestry, Chinese Academy of Forestry, Number 682, Guang Shan Yi Road, Longdong District, Guangzhou, 510520 China

**Keywords:** *Jatropha curcas*, SPL, Genome-wide, miR156, Expression patterns, Age development

## Abstract

**Background:**

SPL (SQUAMOSA-promoter binding protein-like) proteins form a large family of plant-specific transcription factors that play essential roles in various aspects of plant growth and development. They are potentially important candidates for genetic improvement of agronomic traits. However, there were limited information about the *SPL* genes in *Jatropha curcas*, an important biofuel plant.

**Results:**

In Jatropha, 15 *JcSPL* genes were identified. Phylogenetic analysis revealed that most of the JcSPLs were closely related to SPLs from woody plant rather than herbaceous plant and distantly related to monocotyledon SPLs. Gene structure, conserved motif and repetitive sequence analysis indicated diverse and specific functions of some *JcSPL* genes. By combination of target prediction and degradome sequencing analysis, 10 of the 15 *JcSPLs* were shown to be targets of JcmiR156. Quantitative PCR analysis showed diversified spatial-temporal expression patterns of *JcSPLs*. It is interesting that the expression levels of *JcSPL3* were the highest in all tissues examined in 7- or 10-year-old plants and exhibited increasing trend with plant age, suggesting its important role in the regulation of age development in Jatropha. Overexpression of *JcSPL3* in Arabidopsis resulted in earlier flowering time, shorter silique length and reduced biomass of roots.

**Conclusions:**

Through comprehensive and systematic analysis of phylogenetic relationships, conserved motifs, gene structures, chromosomal locations, repetitive sequence and expression patterns, 15 *JcSPL* genes were identified in Jatropha and characterized in great detail. These results provide deep insight into the evolutionary origin and biological significance of plant SPLs and lay the foundation for further functional characterization of *JcSPLs* with the purpose of genetic improvement in Jatropha.

## Background

SPL (SQUAMOSA-promoter binding protein-like) proteins form a major family of plant-specific transcription factors that play important roles in plant growth and development. They include a highly conserved 76 amino acid residue SBP (SQUAMOSA-promoter binding protein) domain. This domain contains two zinc-binding sites essential for DNA binding and a bipartite nuclear localization signal (NLS) at the C-terminal [1, 2]. The SPL genes were first identified in *Antirrhinum majus* for their ability to bind to the floral meristem identity gene *SQUAMOSA* promoter [3]. Ever since, the orthologous SPL genes have been identified in various plants ranging from the single-cell alage (*Chlamydomonas reinhardtii*) [4] and moss (*Physcomitrella patens*) [5], to *Arabidopsis thaliana* [6], rice (*Oryza sativa*) [7], and perennial plant silver birth (*Betula pendula*) [8], apple (*Malus domestica*) [9] and poplar (*Populus trichocarpa*) [10]. In these plants, the SPL genes were uncovered to regulate various aspects including flowering time, leaf development, phase transition, plant architecture, organ size, fruit development and stress response.

The SPL genes exist as a large gene family in plants and can be divided into different groups based on cluster analysis [11]. In Arabidopsis, a total of 16 *SPL* genes were identified and these *AtSPLs* were classified into eight groups [12]. Ten *AtSPLs* including *AtSPL2*–*6*, *AtSPL9*–*11*, *AtSPL13* and *AtSPL15* were targets of miR156 [13–15]. The three small genes *AtSPL3*, *AtSPL4* and *AtSPL5* have a target site for miR156 in their 3′-UTR region, and were found to promote vegetative phase change and flowering [16]. The other group *AtSPL10*, *AtSPL11* and *AtSPL2* control morphological change in association with shoot maturation in the reproductive phase and the lateral root development [17, 18]. Two paralogous genes *AtSPL9* and *AtSPL15* act redundantly in controlling the juvenile-to-adult phase transition [12]. In addition, *AtSPL9* control trichome distribution, sesquiterpene and anthocyanin biosynthesis [15, 18, 19]. *AtSPL13* is essential for the cotyledon-to vegetative-leaf transition [20]. *AtSPL6* positively regulates defense genes in innate immunity [21]. Among the six *AtSPLs* that are not targets of miR156, *AtSPL7* is a central regulator for copper homeostasis [22, 23]. *AtSPL8* is involved in pollen sac development [24], gibberellin signalling [25] and male fertility [26]. *AtSPL14* participates in plant development and sensitivity to fumonisin B1 [27]. The function of three genes *AtSPL1*, *AtSPL12* and *AtSPL16* remain unknown and need further investigation.

Furthermore, the SPL genes are potentially important candidates for genetic improvement of agronomic traits. In rice, a point mutation in *OsSPL14* could generate an ideal rice plant with a reduced tiller number, increased lodging resistance and enhanced grain yield [28]. Manipulation of *BrpSPL9* from *Brassica rapa ssp. pekinensis* optimized the earliness of heading time in Chinese cabbage [29]. Suppression of *PvSPL2* from *Panicum virgatum* increased biomass yield and reduced lignin accumulation and thereby elevated the total amount of solubilized sugars [30]. Overexpression of *rSPL10* gene in *Pogostemon cablin* increased the essential oil content and accelerated plant growth [31].

*Jatropha curcas* L*.* is a potential biofuel plant for sustainable environmental development. The seeds contain an average of 34.4% oil that can be processed to produce a high-quality biodiesel fuel [32]. Given that overexpression of specific SPL gene could accelerate leaf initiation rate, increase biomass yield, enhanced salt tolerance and essential oil content, we ask if SPL is involved in the production of oil in *J. curcas*. There have been several studies regarding the identification of miRNA from *J. curcas* [33–35]. In Jatropha seeds, a total of 24 miR156 family were reported, whereas little information is available about the miR156 target-SPL and the interaction between miR156 and SPL. miR156 has been shown to be the master regulator of vegetative development and stress response by inhibiting the expression of SPL genes [36]. Considering the vital role of miR156/SPL modules in regulation of plant development and growth, we performed a genome-wide investigation of *SPL* genes in *J. curcas.* Some of the 15 identified *SPL* genes were highly conserved based on gene structure, conserved motifs, as well as clustering level. Expression analysis showed that the expression level of *JcSPL3* increased with increasing age. These results provide insights into the biological functions of *SPL* genes in *J. curcas.*

## Results

### Phylogeny of plant SPL family proteins

**Fig. 1:**
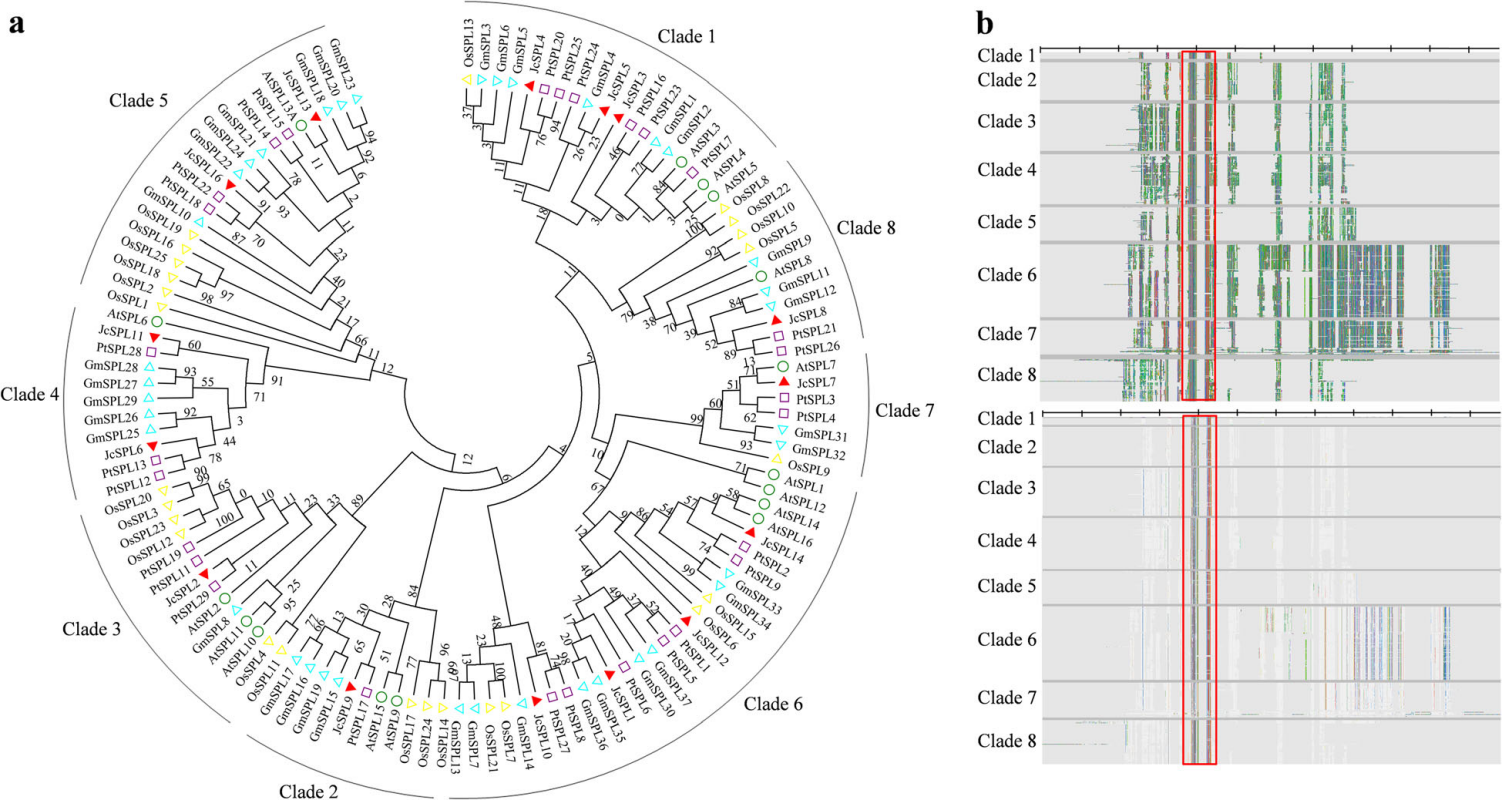
Phylogenetic and structure analysis of plant SPL proteins. **a** Phylogenetic relationship analysis of whole plant SPL proteins. Phylogenetic tree was constructed from 115 SPL proteins using the Maximum-likehood method in MEGA 7. **b** Overall structure analysis of plant SPL proteins. The alignment was marked in AliView to show all amino acids (upper) or majority rule consensus residues (lower). The red box represents the SBP domain

To gain an understanding of the evolutionary status of SPLs from *J. curcas* in all plants, putative SPL family proteins were originally extracted from the Pfam database by using the conserved SBP domain (PF03110) as query. After filtering redundant and short sequences, 833 SPL proteins were obtained from 60 different species (Additional file 1). The largest number of SPL sequences were found in *Musa malaccensis* with 53 sequences, and Chlorophyta contained the least number of SPL, with 1 to 4 sequences. The results showed that SPLs existed as a middle-sized gene family in green plants. These plant SPL proteins could be classified into eight clades. Each clade contained at least one AtSPL protein. Clade 1–5 contained previously well reported AtSPL protein families. Among the groups, clade 6 contained the largest number of sequences with 195 SPL proteins, while clade 1 contained the least number with 33 SPL proteins. However, the function of SPLs in clade 6 were mostly unknown. These suggested that the majority of SPL genes are still unexplored and worth of further functional investigation. There were 15 SPL proteins found from the Pfam database in Jatropha species. To reveal the possible roles of *JcSPLs*, a phylogenetic tree was constructed based on *OsSPLs*, *GmSPLs* (*Glycine max*), *AtSPL*s and *PtSPL*s (Fig. 1a). The results showed that they were divided into eight groups and all *JcSPLs* were grouped together with their orthologous Arabidopsis counterparts except *JcSPL16* and *JcSPL11.* Among them, *AtSPL7* and *JcSPL7*, *AtSPL8* and *JcSPL8*, *AtSPL9* and *JcSPL9*, *AtSPL6* and *JcSPL6*, *AtSPL13* and *JcSPL13* were likely to be orthologous genes. Three pairs of *JcSPLs* including *JcSPL13*/*16*, *JcSPL6*/*11* and *JcSPL1*/*12* shared high sequence similarity and were presumed to be paralogous genes. Furthermore, most of the *JcSPL* genes showed a closer relationship with woody plant Glycine and Populus SPL genes rather than herbaceous plant Arabidopsis SPL genes, and *JcSPL* genes were rather distantly related to the rice SPL genes. These suggested that *SPL* genes could originate from a common ancestor and had undergone divergent differentiation after the separation of each lineage.

Overall structure analysis of all plant SPL proteins revealed that clade 1 were the smallest of the SPL proteins, while clade 6 and clade 7 were the largest (Fig. 1b). Proteins in clade 2–5 and clade 8 had similar protein size. Sequence conservation analysis showed that the SBP domain with approximately 78 amino acid residues were highly conserved across all clades, indicating that they could all bind to the promoters of floral meristem identity gene *SQUAMOSA* and its orthologous genes. The diversity of SPL clades suggested that they were involved in regulating different aspects of plant growth and development besides flowering control.

### Identification of SPL genes in Jatropha

To identify the SPL genes in the Jatropha genome, the SBP domain was used as a query to search against the Jatropha genome. There was a total of 20 full-length SPL genes identified. By combination of both the Pfam database and genome data, the Jatropha proteins were then named based on known Arabidopsis homologues. After removing the splice variants, there were finally 15 loci in Jatropha (Additional file 2). Nevertheless, when we tried to clone one SPL gene (ID: XM_012236245.2) from Jatropha cDNA library, there was a point mutation (A343T) in the coding region which lead to premature termination of protein translation (Additional file 3). Therefore, we kept its splice variant (XM_012236246.2) for further analysis and that was named as *JcSPL3*. Comparing to the 16 *AtSPLs* from Arabidopsis [1], no homologue for *AtSPL15* was found in Jatropha. Among the 15 *JcSPL* genes, *JcSPL13* was in clade 5, four genes *JcSPL1*, *JcSPL12*, *JcSPL14* and *JcSPL10* were classified in clade 6, and *JcSPL7* and *JcSPL8* were in clade 7 and clade 8, respectively. The group member in clade 5, 6, 7 and 8 were consistent with those of *AtSPLs.* The smallest protein was JcSPL3 (142 aa) with a molecular weight (MW) of 16.1 kDa (Additional file 2), while the largest protein was JcSPL14 (1068 aa) with a MW of 118.6 kDa. The theoretical pI of JcSPL proteins ranged from 6.10 (JcSPL7) to 9.44 (JcSPL16). The protein length, MW and pI of JcSPL proteins were similar to PtSPL proteins from Populus [10].

**Fig. 2:**
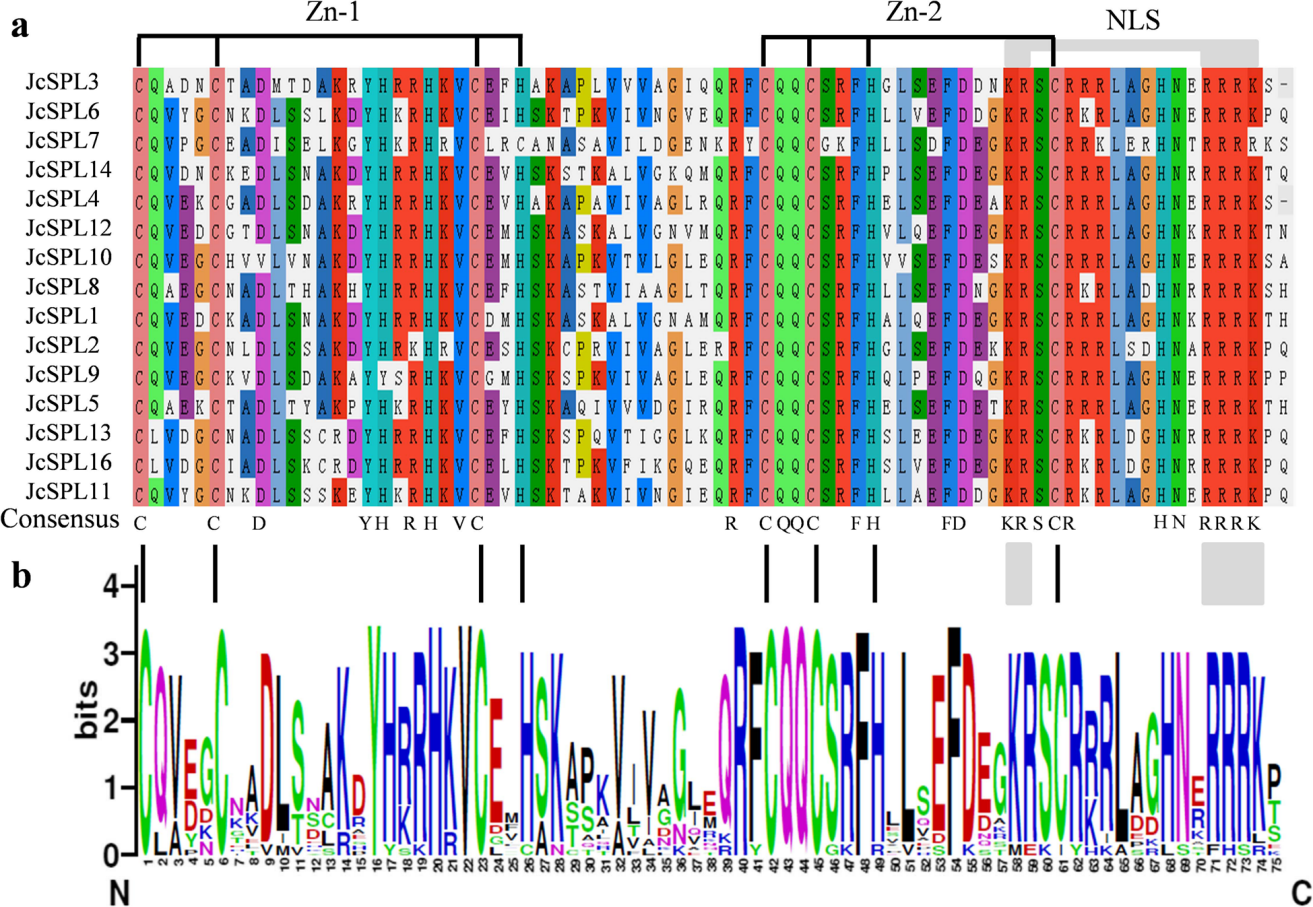
Sequence alignment and logo of the *JcSPLs*. **a** Multiple alignment of the *JcSPL*s. **b** Sequence logo of the SBP domain from the *JcSPLs*

Multiple sequence alignment of all JcSPLs *s*howed the SBP domain were highly conserved at certain positions (Fig. 2a). They contained three conserved domains, including zinc finger 1 (Zn1), zinc finger 2 (Zn2), and bipartite nuclear localization signal (NLS) (Fig. 2b). The Zn1 (Cys_3_His-type) in JcSPL7 was replaced with Cys_4_ signature sequence, which type was also found in Arabidopsis and Populus. The Zn2 (Cys_2_HisCys-type) was the same in all JcSPLs. The NLS was located at the C-terminus of SBP domain with KRRRR signature sequence that partly overlapped with Zn2 structure. The SBP domain organization was highly conserved among moss [1], Arabidopsis and Populus, indicating the SBP domain organization was anciently established in plants.

The 15 *JcSPL* genes were further mapped onto the 11 linkage groups (LGs) of *J. curcas* [37]. These *JcSPL* genes were unevenly distributed across seven LGs (Additional file 4), with four genes on LG8, and three genes on LG1 and LG7. LG5 contained two *JcSPL* genes, while LG3, LG4 and LG6 only displayed one *JcSPL* gene, respectively. The majority of *JcSPL* genes were located on the top and bottom regions on LG1, LG3, LG4, LG6, LG7 and LG8. Two genes *JcSPL13* and *JcSPL11* were on the middle part of LG5. The synonymous (Ks) and non-synonymous (Ka) substitution rates ratios (Ka/Ks) for six pairs of *JcSPLs* were less than 1.0, indicating purifying selection (Additional file 5).

### Gene structure, motif and sequence analysis of *JcSPL* genes

**Fig. 3:**
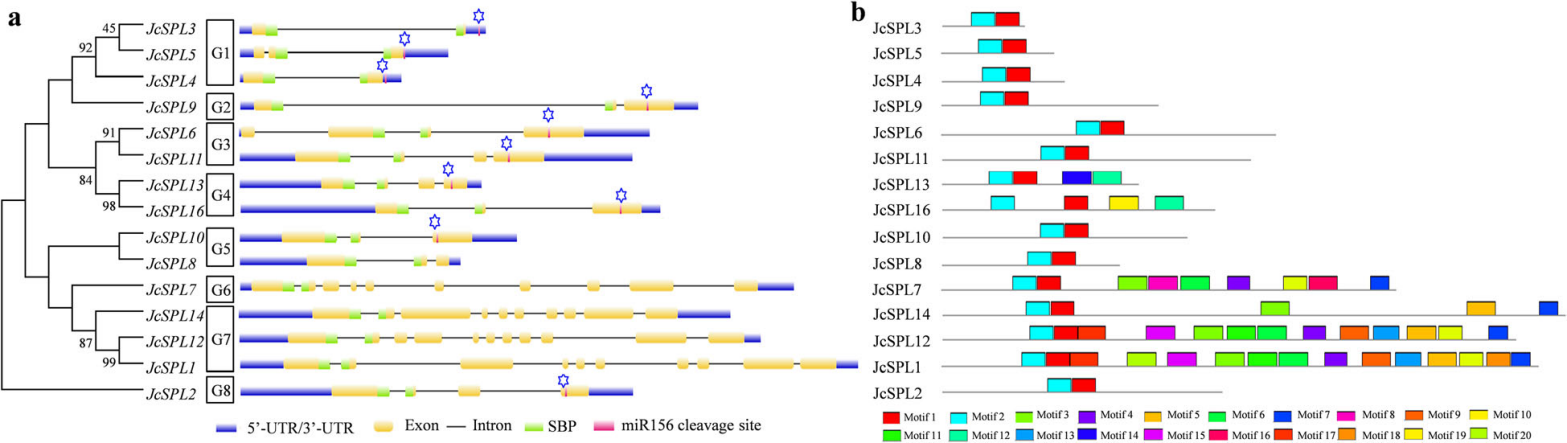
Gene structure and motif analysis of the *JcSPLs*. **a** Phylogenetic tree and gene structure of *JcSPLs*. The tree was constructed using the 15 JcSPL protein sequences. The asterisk indicates the miR156 cleavage site. **b** Distribution of the conserved motifs in the *JcSPLs*

To provide further insight into the evolutionary relationships of *JcSPL* genes, the full-length JcSPL proteins were used to construct a phylogenetic tree. It showed that they were clustered into eight subgroups (Fig. 3a). The gene structure analysis revealed that the conserved SBP domain were interrupted by the first intron in all 13 *JcSPLs* except *JcSPL5* and *JcSPL6*, in which the SBP domain were interrupted by the second intron. The position of intron in the SBP domain were highly conserved and located in the 47th amino acid, which were also found in Arabidopsis [6] and Populus [10]. However, the intron length varied greatly with the range from 86 bp in *JcSPL7* to 3967 bp in *JcSPL9*. The closely related members within the same group usually shared similar exon/intron structures in terms of length and number. The intron number existing in the 15 *JcSPLs* ranged from 1 to 10. The *JcSPL* genes in Group 1 were the smallest and had only one or two introns, while *JcSPL* genes in Group 7 were the largest and included eight to ten introns. The other *JcSPL* genes had two or three introns. The intron number of *JcSPLs* were similar to those of *AtSPLs, PtSPLs* and *CclSBPLs* from *Citrus Clementina* [10, 38], suggesting the conservation of SPL gene structures among plants.

The detailed length of exon and intron were further analyzed (Additional file 6). It was found that the exon length of *JcSPLs* ranged from 77 to 850 bp with an average of 290 bp, which were similar to those of *AtSPLs* with an average of 297 bp. The intron length of *JcSPLs* ranged from 55 to 3966 bp with an average of 482 bp, while the largest intron length in *AtSPLs* was only 648 bp and the average was 124 bp. It could be concluded that, the exon size distribution of *JcSPLs* were similar to those of *PtSPLs* and *AtSPLs*, while the intron size distribution differed greatly among three of them [10]. These indicated the important role of intron in plant evolution.

Further analysis of conserved motifs of JcSPL family were performed. There was a total of 20 motifs identified for the 15 JcSPL proteins (Fig. 3b, Additional file 7). The number of motifs in each JcSPL varied from 2 to 15. Motif 1 and motif 2 (SBP domain) were found in all JcSPLs. Most closely related members contained similar motif types and number. Moreover, Motif 12 was only found in JcSPLs from Group 4, and motif 14 and motif 16 was unique in JcSPL13 and JcSPL7, respectively. In addition to the conserved SBP domain, other conserved motifs including motif 13 and motif 18 which correspond to the ANK domain were found in Jatropha, Salvia and Arabidopsis SPLs [39]. The diversity and specificity of motifs among these JcSPLs indicated the diverse and specific functions of JcSPLs.

**Fig. 4:**
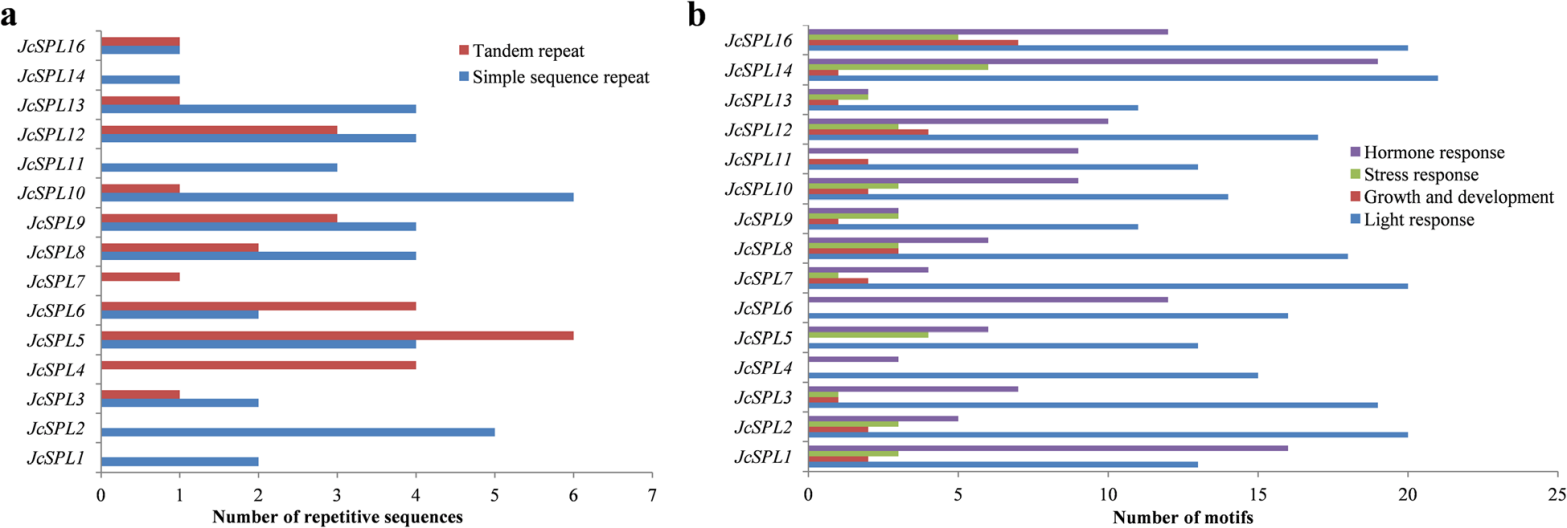
Distribution of repetitive sequences and motifs in the *JcSPLs*. **a** The number of repetitive sequences in the promoter and genomic DNA sequences of *JcSPLs*. **b** The number of cis-acting elements found in the promoter regions of *JcSPLs*

The 2.0 kb sequence upstream of each *JcSPL* gene were then retrieved as promoter for repetitive element and *cis*-element analysis. It was found that all *JcSPLs* showed repetitive sequence features including either simple sequence repeat (SSR) or tandem repeat (TR) in the promoter and gene regions (Fig. 4a). The SSR markers occurred more frequently than TR, which were similar to *CclSBPLs* [38]. The SSR were found in all *JcSPLs* except *JcSPL4* and *JcSPL7*, while TR were found in 11 *JcSPLs*, among which *JcSPL5* had six TR sequences.

SPL genes belong to transcription factors and they were also tightly regulated at the transcriptional level [13]. The *cis*-acting elements in the promoter regions of all *JcSPLs* were investigated (Fig. 4b). The CAAT-box and TATA-box that were core promoter element were found in all *JcSPL* genes (Additional file 8). The *cis*-acting elements involved in the light response were the most abundant, followed by hormone response including abscisic acid, methyl jasmonate, salicylic acid, gibberellin and auxin responsiveness, and then stress response [40]. The putative elements involved in growth and development and stress response were absent in the promoter regions of *JcSPL4* and *JcSPL6*. There were seven and four *JcSPL* genes containing the CAT-box and GCN4 motif, which were functional in the meristem and endosperm expression, respectively. Besides, the promoter of *JcSPL16* showed elements involved in flavonoid biosynthesis and cell cycle regulation, while *JcSPL2* had elements involved in circadian control. These suggested that *JcSPLs* could participate in various physiological and developmental regulation.

### Posttranscriptional regulation of *JcSPLs*

**Fig. 5:**
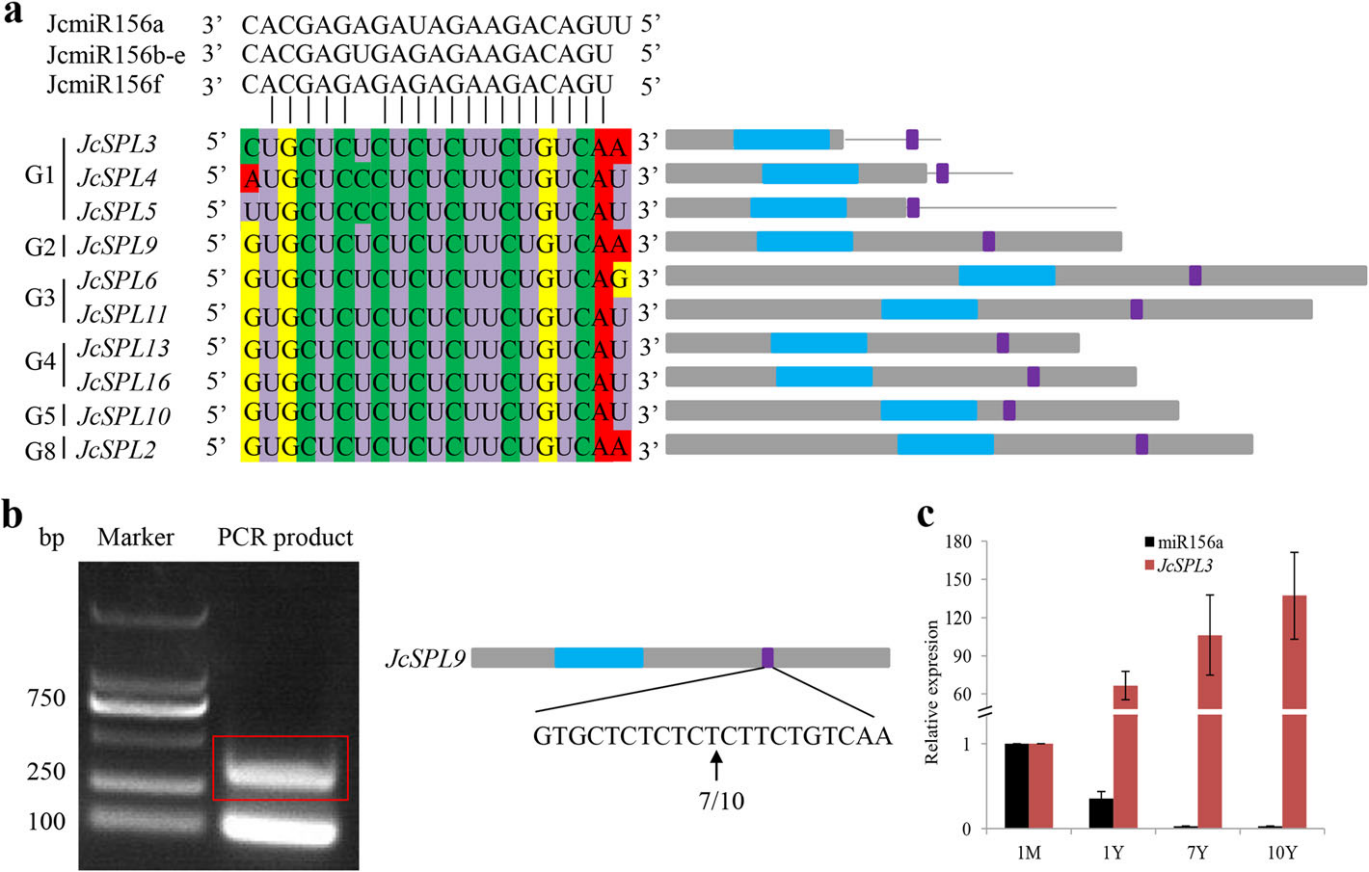
Target sequences prediction and RLM 5′-RACE validation of *JcSPLs* cleaved by the miR156 and expression of miR156. **a** Target sequences of 10 *JcSPLs* through psRNATarget prediction. Left are the multiple alignment of miR156 complementary sequences with their targets. Right are the gene structure of corresponding *JcSPLs*. Blue box indicates SBP site. Purple box indicates the miR156 target site. **b** RLM-5′-RACE validation of *JcSPL9* cleavage sites by JcmiR156a. The red box indicates PCR products for RLM 5′-RACE. The arrow indicates cleavage sites verified by RLM 5′-RACE with the sequencing frequency (sequencing reads/total sequenced clones) of cloned PCR products. **c** Relative expression of miR156a and *JcSPL3*. The expression levels at 1-month-old plants were set as 1.0. The value was shown as the mean ± the standard deviation (*n* = 3)

Several members of SPL family were reported to be post-transcriptionally regulated by miR156 in different plants [13, 28]. In order to get deep understanding of the functional roles of *JcSPL* genes in Jatropha, we evaluated the potential regulation of *JcSPLs* by miRNA through both psRNATarget prediction and degradome sequencing. The mature miR156 was found to be present in Jatropha seeds by small RNA sequencing reported earlier [34]. Six miR156 genes (JcmiR156a-f) that exhibited obvious expression levels were identified and were used for further analysis. The MFold-predicted secondary structure of the miR156 precussor sequence showed a hairpin loop with mature miR156 in its stem region, a characteristics of miRNA precussor (Additional file 9). Then we used the six miR156 sequences and psRNATarget to predict the potential targets in all 15 *JcSPLs*. The results showed that 10 *JcSPLs* were identified as targets of JcmiR156 (Fig. 5a). Most miR156-targeted *JcSPL* genes were clustered into Group 1–4 (Fig. 3a). The JcmiR156 target sites for three *JcSPLs* in Group 1 were located in the 3′ UTRs, while the target sites for all the other *JcSPLs* were in their last exons.

Furthermore, degradome sequencing approach were applied to validate the miR156-mediated regulation of *JcSPLs*. A mixture of nine samples including leaf, stem and root tissues from 1-month-, 7-year- and 10-year-old plants, respectively, were used for library construction. The acquired sequences were matched to Jatropha genome assembly JatCur_1.0 [36]. After analysis, a total of 9 *JcSPL* transcripts were identified to be cleaved by miR156 and miR157 family genes (Additional file 10). These *JcSPL* genes were consistent with psRNATarget prediction, except that *JcSPL4* was not found, which may be due to the relatively low expression levels of this gene (Additional file 11). Among the 9 *JcSPL* targets, 8 *JcSPLs* were identified to be cleaved by 19 miR156 family genes and 3 miR157 family genes, while *JcSPL5* were targets of 14 miR156 family genes and one miR157 gene. Considering that miR157 shares 14 nt to 16 nt sequence similarity with miR156 and there was no previous report about miR157-mediated cleavage of SPL, we concluded here that the identified 9 *JcSPLs* were predominantly regulated by miR156. The cleavage sites in the *JcSPL* gene sequences were clearly shown in T-plots and were confirmed to be exactly the same as predicted sites (Additional file 12). The target member distribution in clades and cleavage site distribution in targets were similar to those of *PtSPLs* and *AtSPLs*, suggesting miR156-mediated regulation of SPLs were highly conserved across plants.

In addition, the cleavage site of *JcSPL9* was validated by 5′ rapid amplification of cDNA ends (RACE) experiments (Fig. 5b). The expression of miR156 was investigated by stem-loop PCR. The results showed that the expression level of miR156 decreased significantly with plant age (Fig. 5c), while *JcSPL3* exhibited an opposite trend of expression.

### Expression patterns of *JcSPL* genes

**Fig. 6:**
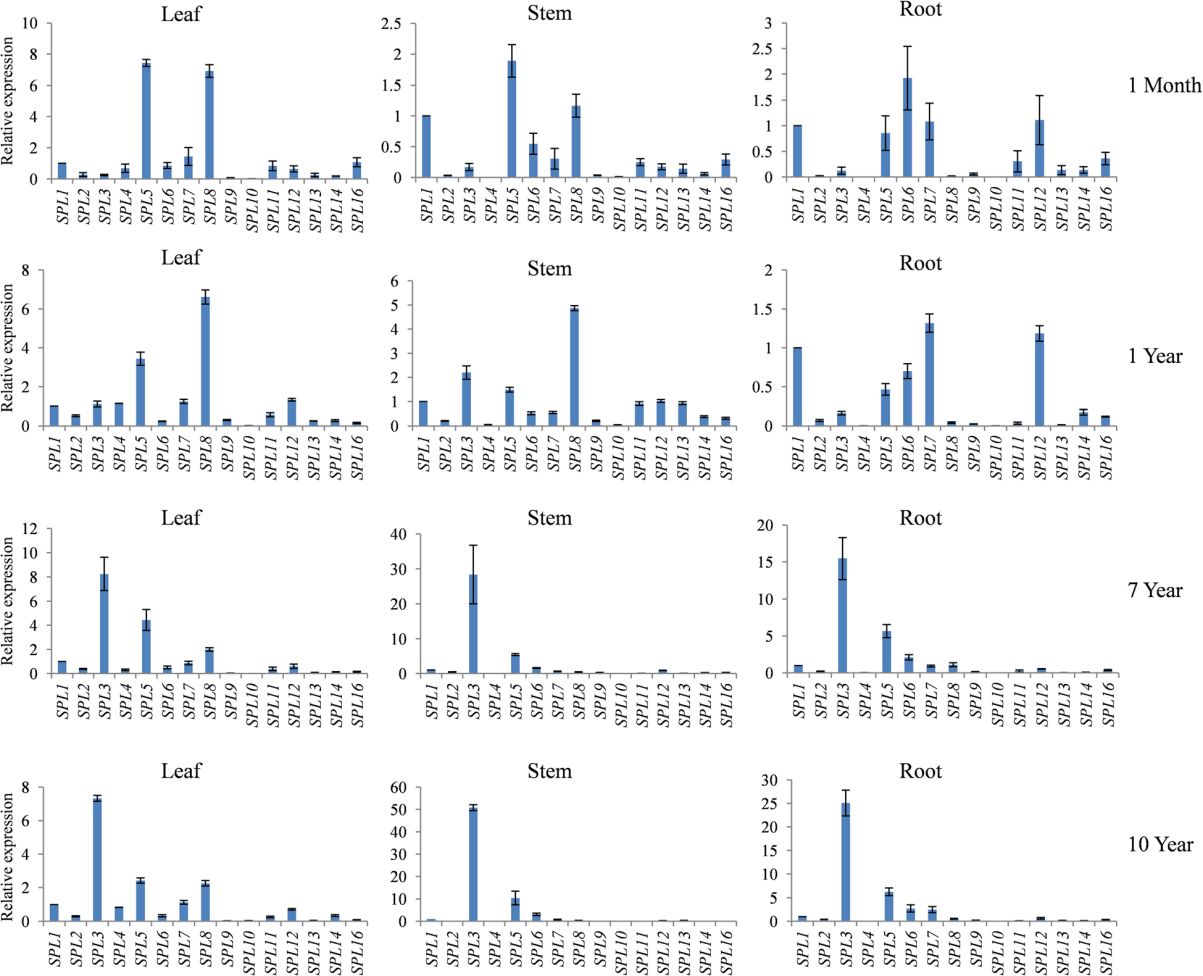
Expression patterns of the *JcSPLs* in different tissues. The expression levels of *JcSPLs* were normalized to the levels of *JcSPL1* in each tissue. The *JcUBQ* gene was used as an internal control. The value was shown as mean value from three biological replicates. Error bars represent the standard deviation

In Jatropha, there were dramatic variability in the leaf morphology from different ages of plants (Additional file 13). The 1-month-old plants produced small and deltoid leaves, while the leaves of 1-year-old trees were five-lobed in shape. The expression patterns of genes could indicate their biological functions. To determine the spatial-temporal expression patterns of *JcSPL* genes, qRT-PCR experiments were performed in various tissues including leaf, stem and root from 1-month-, 1-year-, 7-year- and 10-year-old trees. In general, all *JcSPL* genes exhibited tissue-specific and age-specific expression patterns (Fig. 6). In particular, *JcSPL5* and *JcSPL8* showed much higher expression levels than all other *JcSPL* genes in both leaf and stem tissues from 1-month- or 1-year-old plants, whereas *JcSPL3* showed the highest transcript abundance than all other *JcSPL* genes in leaf, stem and root tissues from 7-year- or 10-year-old plants. Furthermore, *JcSPL10* exhibited lower expression levels than other *JcSPL* genes in all tissues from 1-month- or 1-year-old trees, whereas eight *JcSPL* genes (*JcSPL2/9/10/11/12/13/14/16*) were expressed at lower levels than other *JcSPL* genes in all tissues from 7-year- or 10-year-old trees.

**Fig. 7:**
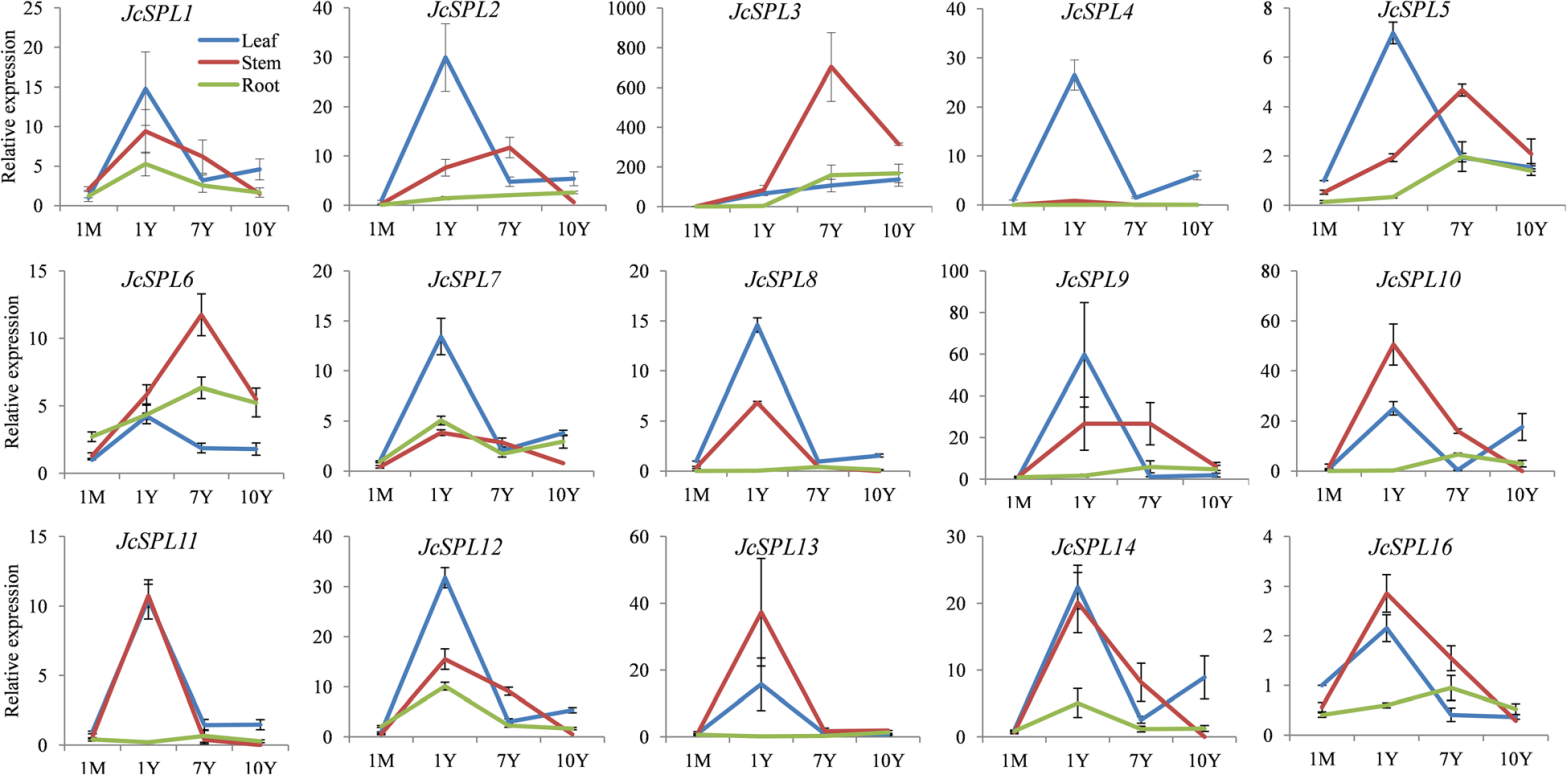
Expression patterns of the *JcSPLs* in different developmental stages. The expression levels of *JcSPLs* in each stage were normalized to the levels in 1-month-old leaf tissue. The *JcUBQ* gene was used as an internal control. The value was shown as mean value from three biological replicates. Error bars represent the standard deviation

The expression patterns of some genes exhibited significant trends during different developmental stages in certain tissues (Fig. 7). Generally, the expression patterns of *JcSPLs* can be divided into three categories. The first and second categories included genes whose expression peaked in 1-year-old and 7-year-old tissues, respectively, while the third category contained genes whose expression in tissues increased as the plants age. In particular, all *JcSPL* genes except *JcSPL3* exhibited higher expression levels in 1-year-old leaf tissue than those from leaf at other stages. It was worth noting that, the expression levels of *JcSPL3* were consistently increased along with the plant age development in leaf tissue, suggesting its role in regulation of age development. Ten *JcSPL* genes (*JcSPL1/4/7/8/10/11/12/13/14/16*) showed higher expression in 1-year-old stem tissue compared to stem from other stages, while other four *JcSPL* genes (*JcSPL2/3/5/6*) showed preferential expression in 7-year-old stem tissue compared to stem from other stages. There were eight *JcSPL* genes (*JcSPL4/5/6/8/9/10/11/16*) that showed higher expression in 7-year-old root tissue compared to root from other stages, while four *JcSPL* genes (*JcSPL1/7/12/14*) showed higher expression in 1-year-old root tissue compared to root from other stages. The other three genes *JcSPL2*, *JcSPL3* and *JcSPL13* showed consistent increasing trend with the plant age development in root tissue, suggesting their specific role in plant root development. In particular, three genes *JcSPL1, JcSP12* and *JcSPL14* belonging to the same subgroup showed similar expression patterns in all samples examined, implying their functional redundancy. However, other *JcSPL* genes even in the same subgroup exhibited diversified expression patterns, indicating that they could play specific roles in various tissues under different developmental stages.

With regard to the interesting expression patterns of *JcSPL3*, the subcellular localization of JcSPL3 protein was further examined. Confocal microscopy observation of the JcSPL3-GFP florescence signals showed that JcSPL3 were mainly distributed in the nucleus (Additional file 14), indicating its potential binding ability in vivo.

### Functional analysis of *JcSPL3*

**Fig. 8:**
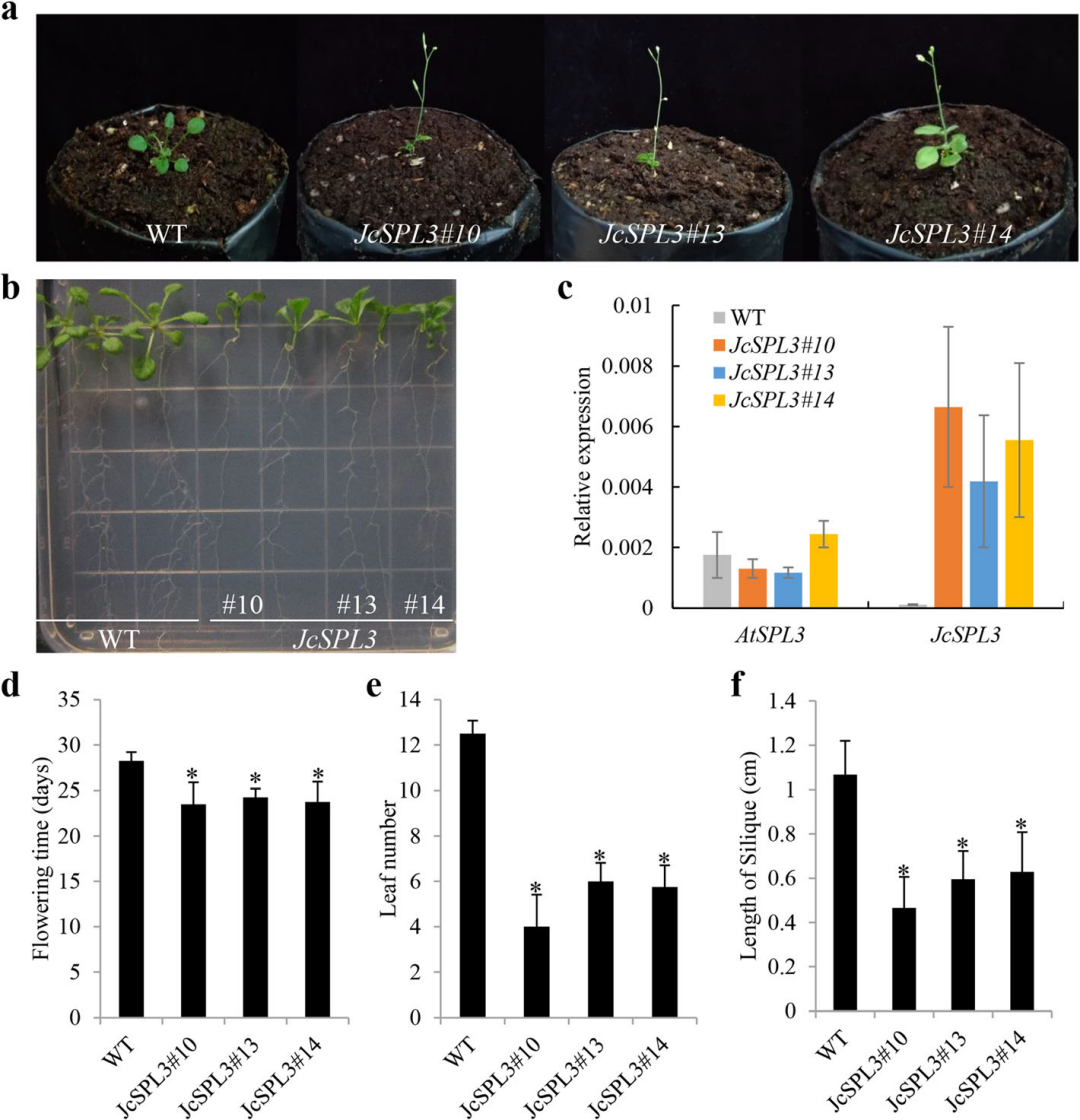
The phenotype of transgenic Arabidopsis with *JcSPL3*. **a** Flowering phenotype. **b** Root phenotype. **c** Expression levels of *AtSPL3* and *JcSPL3*. **d** Flowering time. **e** The number of rosette leave at the time of flowering. **f** The length of silique

*JcSPL3* exhibited an obvious increasing trend with plant age development and we speculated that this gene may be important for the growth and development of Jatropha. Therefore, the function of *JcSPL3* was investigated by overexpression in Arabidopsis (Fig. 8). Three independent transgenic lines were selected for phenotypic observation. The plants overexpressing *JcSPL3* flowered significantly earlier than the wild-type plants in terms of both flowering time and the number of rosette leaves to flowering (Fig. 8a), and produced fewer lateral roots than wild-type (Fig. 8b). The expression level of *JcSPL3* in the overexpressed lines was about 50 times more than the wild-type (Fig. 8c). The average time to flowering of the transgenic plants ranged from 23.5 to 24.3 days, while that of the wild-type plants was 28.3 days (Fig. 8d). The average number of rosette leaves at flowering ranged from 4 to 6 in the transgenic plants, and was 12.5 in the wild-type plants (Fig. 8e). In addition, the siliques of transgenic plants were approximately 50% as long as those of the control plants (Fig. 8f). These data indicated that *JcSPL3* may act as a floral activator and might be involved in the Jatropha flowering process.

## Discussion

SPLs are plant-specific transcription factors that play significant regulatory roles in plant growth and development. Since its discovery in *A. majus*, the orthologous *SPL* genes have been identified in algae, moss, Arabidopsis and crops. However, less study is reported from perennial woody plants [8, 10]. In this study, the evolutionary status of Jatropha SPL genes was initially investigated. After filtering the short and partial sequences in the Pfam database, a total of 833 full-length SPL genes were found from 60 species which represent plant lineages of green algae, moss, lycopodiophyta and angiosperm. However, no SPL genes were discovered in the Pfam database from gymnosperms. To our knowledge, there were limited papers reporting about the identification and functional analysis of SPL genes from gymnosperm [5, 38]. More information about SPLs from gymnosperm would further help clarify the evolutionary process of plant SPLs.

Phylogenetic analysis of plant SPLs divided them into eight groups (Fig. 1). Among these groups the relatively small SPL genes were clustered in clade 1, whereas the relatively large SPL genes were distributed in clade 6. Moreover, the miR156-targeted SPL genes, based on the information from Arabidopsis and Jatropha, were distributed into clade 1–5. In addition, the orthologous genes from different species were more closely related than their members in other groups from the same species. These all indicated that the SPL genes may originate from a common ancestor of green plants.

In Jatropha genome, there were 15 *JcSPL* genes identified, among which five *JcSPLs* contained splice variants (Additional file 2). To verify the sequences of *JcSPL* genes retrieved from the *J. curcas* genome database [36], we cloned and sequenced these *JcSPLs*. The cDNA amplification results showed that there was a point mutation in the gene XM_012236245.2 (Additional file 3). To exclude the possibility of single nucleotide polymorphism (SNP) site in this gene among different individuals, we randomly chose five plants and extracted their DNA for amplification. The PCR sequencing results showed that the gene sequences were consistent with those obtained from cDNA library. Therefore, we speculate that this nucleotide error was caused by whole genome sequencing. In our study, the other splice variant *JcSPL3* was chosen for further analysis. The number of *JcSPL* genes identified in Jatropha was similar to that in Arabidopsis, but was significantly less than that in Populus, apple and maize [7, 9, 10]. In those plants the *SPLs* have undergone gene duplications. This relatively small number of gene family was also reported for other transcription factor families in the Jatropha genome, such as the WRKY gene family, which have not undergone any recent gene duplication [36]. In Arabidopsis, four pairs of 16 *AtSPL*s (*AtSPL10*/*11*, *AtSPL9*/*15*, *AtSPL1*/*12* and *AtSPL14*/*16*) were likely to be paralogous genes. The number of homologous *JcSPL* gene pairs was similar to that in Arabidopsis and *C. clementina*, indicating that SPL genes were conserved throughout plant genome.

Based on the phylogenetic relationship, the 15 *JcSPLs* were classified into eight groups (Fig. 3), which was similar to the phylogenetic tree of *AtSPLs* [12], but inconsistent to the classification of *PtSPLs* [10] and *CclSPLs* [38]. This inconsistency may be due to different method of phylogenetic construction. According to the phylogenetic tree, three pairs of *JcSPL* genes were presumed to be segment duplicated genes. It also revealed that *JcSPLs* were more closely clustered with Populus *SPLs* rather than *AtSPLs* (Fig. 1) and most *JcSPLs* were closely grouped together with their orthologous Arabidopsis counterparts. For example, *JcSPL3/4/5* were clustered together with *AtSPL3/4/5* in one group. Moreover, *JcSPL3/4/5* were relatively small and had a miR156 target site in the 3′ UTR, which were also similar to *AtSPL3/4/5,* suggesting that *JcSPL3/4/5* may have similar function in regulating flowering time and phase transition in Jatropha.

Gene structure and conserved motifs analysis revealed that members in the same group always showed similar structure and motifs (Fig. 4), suggesting possible redundant function of these *JcSPLs*, such as *JcSPL3*/*4*/*5* and *JcSPL1*/*12*. The number of conserved motifs in *JcSPLs* was more than those in *AtSPLs*, indicating that some *JcSPLs* may have species-specific role. Moreover, there were about two-thirds of the *JcSPLs* contained the miR156 target sites and nine *JcSPLs* were confirmed by degradome sequencing to be cleaved by miR156 (Additional file 12). The complementary site to miR156 in the three small *JcSPLs* in Group 1 located in the 3′-UTR region, whereas in other seven *JcSPLs* it located in the last exon. This is also found in SPL genes of other plants including Arabidopsis and Populus, indicating the conservation of miR156-mediated regulation of SPLs in plant kingdom.

Expression pattern analysis revealed that most *JcSPLs* exhibited differential expression in various tissues and developmental stages (Figs. 6 and 7), indicating diverse function of *JcSPLs* in Jatropha growth and development. Previous reports have shown that most of paralogous *SPL* genes in the same group exhibited similar expression patterns [10, 38]. In Jatropha, three genes *JcSPL1*, *JcSPL12* and *JcSPL14* belonging to Group 7 showed similar expression patterns, whereas other genes even in the same group showed distinct expression, suggesting specific functions of these genes. Interestingly, the expression levels of *JcSPL3* increased significantly with the age of the plant in both leaf and root tissues (Fig. 7). Studies have shown that *AtSPL3* is responsible for plant phase transition and this transition is conserved in both annual herbaceous plants and perennial plants [2, 41, 42]. In Jatropha, there was a significant variability in the morphology of the leaves from 1-month- and 1-year-old shoots of plants. In our study, the expression of *JcSPL3* increased significantly in leaf from 1-year-old plants compared to that from 1-month-old plants. These together strongly suggest that *JcSPL3* may be responsible for the vegetative phase transition in Jatropha. This was confirmed by overexpression of Jatropha *SPL3* into Arabidopsis, which revealed earlier flowering phenotype. In addition, the levels of *JcSPL3* also showed increasing trend in roots with increasing age. The roles of *SPLs* in control of lateral root development were previously characterized [18, 43]. These suggest that *JcSPL3* could also play a role in the root development (Fig. 8b). Together, these results in our study uncover the functional roles of Jatropha *SPL* genes and provide important candidates for further functional characterization with the aim of genetic improvement of growth and development traits.

## Conclusions

The phylogeny of plant SPL proteins from the PFAM database were performed. Through comprehensive and systematic analysis of phylogenetic relationships, conserved motifs, gene structures, chromosomal locations, repetitive sequence, posttranscriptional regulation, expression patterns and subcellular localization, 15 *JcSPL* genes were identified in Jatropha and characterized in great detail. Interestingly, the expression patterns of *JcSPL3* indicate its important role in the regulation of age development in Jatropha. These results provide deep insight into the evolutionary origin and biological significance of plant SPLs. Moreover, our study provides potential candidates for further functional characterization of *JcSPLs* with an aim of genetic improvement in Jatropha.

## Methods

### Analysis of plant SPL family

All SBP domain (PF03110) containing proteins were identified using the PFAM database (http://pfam.xfam.org/family/SBP#tabview=tab1). The full-length protein sequences were extracted using the uniprot IDs from uniprot database (http://www.uniprot.org/). After removing redundant and short sequences, a total of 833 sequences were obtained. For phylogenetic analysis, a bootstrapped neighbour joining tree was constructed using the MEGA 7. Overall structure analysis was performed in AliView. Sequence conservation logos were generated using the WebLogo platform (http://weblogo.berkeley.edu/logo.cgi).

### Analysis of *JcSPL* genes

Sequences of 16 *AtSPLs* genes were retrieved from the Arabidopsis TAIR database (http://www.arabidopsis.org/). To identify the *JcSPL* genes, BLAST search against *J. curcas* genome assembly JatCur_1.0 were carried out using *AtSPLs* as queries (https://www.ncbi.nlm.nih.gov/genome/?term=Jatropha). All putative proteins were further confirmed to contain SBP domain in the PFAM database. Originally 21 *JcSPL* genes were retrieved. After filtering the splice variants and keeping one representative transcript, 15 *JcSPL* genes were obtained. Molecular weight (Mw) and theoretical isoelectric point (pI) parameters were predicted using the ExPASy program (http://web.expasy.org/protparam/).

Chromosome locations of all *JcSPL* genes were generated using MapChart 2.1 based on the Jatropha linkage map [35]. The ratio between nonsynonymous and synonymous nucleotide substitutions (Ka/Ks) was calculated using KaKs_Calculator 2.0 for selected pairs of homologous genes [44].

The exon/intron structure analysis of *JcSPL* genes were performed with the Gene Structure Display Server (http://gsds.cbi.pku.edu.cn/). Protein conserved motifs were predicted using the MEME program (http://meme-suite.org/tools/meme). An E-value cut off of Ie^− 10^ was used.

The repetitive sequence analysis in the promoter (2.0 kb upstream of the coding sequence) and coding sequence of *JcSPLs* were determined by SSRIT database (http://archive.gramene.org/db/markers/ssrtool) and Tandem Repeat Finder database (https://tandem.bu.edu/trf/trf.html). The *cis*-acting elements analysis in the promoter region of all *JcSPLs* genes were performed by using the PlantCARE database (http://www.dna.affrc.go.jp/PLACE/signalscan. html) [40].

### Prediction of *JcSPLs* targeted by miR156

The sequence of JcmiR156a-f was obtained from the data in the paper by Galli et al. [34]. The identified pre-miRNA secondary structure was predicted using the mfold Web Server (http://unafold.rna.albany.edu/?q=mfold/RNA-Folding-Form). psRNATarget (http://plantgrn.noble.org/psRNATarget/) was used to predict the targets of JcmiR156 by searching the coding regions and 3′-UTRs of all *JcSPLs* for complementary sequences of JcmiR156a-f using default parameters.

### Degradome sequencing

Fully expanded leaves, stem and root were harvested from 1-month-old plants grown in the greenhouse. The fourth leaves from lateral branches, stem with 5 cm length and lateral roots were harvested from 1-year-, 7-year- and 10-year-old plants growing outdoors. Total RNA of nine tissues including leaf, stem and root from 1-month-old, 1-year-, 7-year- and 10-year-old plants were extracted using the Plant Qiagen RNeasy Kit (Qiagen China, Shanghai) following the manufacturer’s instructions. The on-column DNase digestion were used for DNA removal and the RNA integrity was checked on an agarose gel. All RNA samples from nine tissues were pooled together in equal amounts and used for library constructions as described previously with minor modifications [45]. In brief, poly(A)-enriched RNA was ligated to RNA adaptor and the ligated products were used to generate first-strand cDNA by reverse transcription. The cDNA was then amplified for 6 cycles (94 °C for 30 s, 60 °C for 20 s, and 72 °C for 3 min) and ligated to double adaptor. The final cDNA library was purified and used for sequencing with the Illumina HiSeq2000 following the manufacturer’s instruction. After adaptor sequences and low-quality reads were removed, CleaveLand 3.0 pipeline was used to detect potentially cleaved targets, with Jatropha miRNA and mRNA sequences as references. T-plots were generated according to the abundance of the resulting mRNA tags relative to the overall profile of degradome reads that matched the target. All targets were classified into five categories. In category 0, the most abundant tag was the only one degradome tag detected, and it was located at the expected cleavage site. In category 1, there were more maximum positions. In category 2, the abundance of cleavage tags fell between the maximum and median values. In category 3, the abundance of cleavage tags was below the median value. In category 4, only one raw read was matched at the cleavage position of the transcript.

### 5′-race

The RNA Ligase-Mediated (RLM) 5′-RACE experiment was performed following the instructions of the RACE kit FirstChoice™ RLM-RACE Kit (Invitrogen). Gene-specific primers (Additional file 15) were designed to conduct nested PCRs, and PCR products were gel purified, cloned and sequenced.

### Stem-loop reverse transcription (RT) PCR

The relative expression level of miR156 were examined by stem-loop PCR [46]. Briefly, total RNA was extracted and reverse transcription was performed using specific stem-loop RT primers for miRNA (Additional file 15). The Jatropha *UBQ* gene was used as the internal control.

### Quantitative RT PCR (qRT-PCR)

Total RNA was extracted by using the Qiagen plant RNA extraction kit according to the manufacturer’s instructions (Qiagen, Germany). qRT-PCR was performed in triplicates using the SYBR Premix Ex Taq™ II Kit (TaKaRa, Dalian, China) according to the manufacturer’s instructions. Three biological replicates were performed. The Jatropha *UBQ* gene was used as the internal control. The primers were listed in Additional file 15.

### Subcellular localization

The coding sequence of *JcSPL3* was cloned from the leaf cDNA library and fused to C-terminal Green florescence protein (GFP) tag (JcSPL3-GFP) in the expression vector pCambia1301-GFP. The construct was transiently expressed in tobacco leaf epidermal cells according to the protocol described by Wydro et al. [47]. Infiltrated leaves were mounted on slides and imaged using a confocal laser-scanning microscope (Nikon C2-ER) with a standard filter set. The empty GFP vector was used as control.

### Arabidopsis transformation

The full-length CDS sequence of *JcSPL3* was cloned into binary expression vector pCambia2301 and transferred into the Agrobacterium strain GV3101. *Arabidopsis thaliana* (Col) was transformed using the floral dip method [48]. T1 seeds were harvested and selected on MS medium with kanamycin 50 μg/ml. Plants were grown in growth chamber under long-day conditions.

## Supplementary information

**Additional file 1.** Detailed information of the plant SPL proteins used for phylogenetic analysis.

**Additional file 2. **Sequence information of identified *JcSPL* genes.

**Additional file 3. **The predicted gene structure and sequence alignment of *JcSPL3*. **a** Schematic representation of the alternative processing of predicted *JcSPL3* genes. **b** Sequence alignment of predicted *JcSPL3* and cloned genes (Upper), and chromatogram information.

**Additional file 4. **Chromosomal localization of the *JcSPL*s. Chromosomal localization of the *JcSPLs* based on the linkage map. The scale is in centimorgan.

**Additional file 5. **The Ka/Ks ratios of the *JcSPLs* family.

**Additional file 6. **Size distribution of exons and introns in *JcSPLs* and *AtSPLs*. **a** Size distribution of exons in *JcSPLs* and *AtSPLs*. **b** Detailed size distribution of small exons in *JcSPLs* and *AtSPLs*. **c** Size distribution of introns in *JcSPLs* and *AtSPLs*. **d** Detailed size distribution of small introns in *JcSPLs* and *AtSPLs*.

**Additional file 7. **E-value and consensus sequence of twenty motifs identified in the *JcSPLs*.

**Additional file 8. **Detailed information of the *cis*-acting elements in the promoters of *JcSPLs*.

**Additional file 9.** Secondary structure of the JcMIR156 family.

**Additional file 10. **The miR156-mediated cleavage site in 10 *JcSPLs* confirmed by degradome sequencing.

**Additional file 11. **Detailed information of miR156-targeted cleavage of *JcSPLs* by degradome sequencing.

**Additional file 12.** Target plots of the targets cleaved by the miR156 family through degradome sequencing. The T-plots show the distribution of the degradome tags along the full-length of the target mRNA sequence (bottom). The red line represents the sliced target trancripts and is marked with arrow.

**Additional file 13.** Leaf morphology in different ages of plants. Scale bars represents 1 cm.

**Additional file 14. **Subcellular localization of JcSPL3 protein. Confocal laser scanning microscopy of JcSPL3 using GFP-fusion proteins in *Nitotiana benthamiana*. Merged indicates combined GFP fluorescene and chlorophyll autofluorescene. Scale bars = 20 μm.

**Additional file 15.** Primers used in this study.

## Data Availability

The datasets used and analyzed during the current study are available in the manuscript and its additional files.

## Abbreviations

NLS: Nuclear localization signal
LG: Linkage groups
SSR: Simple sequence repeat
TR: Tandem repeat
SBP: SQUAMOSA-promoter binding protein
PCR: Polymerase chain reaction
qRT-PCR: Quantitative real-time reverse transcription PCR
GFP: Green florescence protein
