# Supplementary figures and images for: Genome-wide characterization of the SPL gene family involved in the age development of Jatropha curcas
Source: BMC Genomics. 2020 May 20;21:368. doi: 10.1186/s12864-020-06776-8 (PMC7238634; doi:10.1186/s12864-020-06776-8)

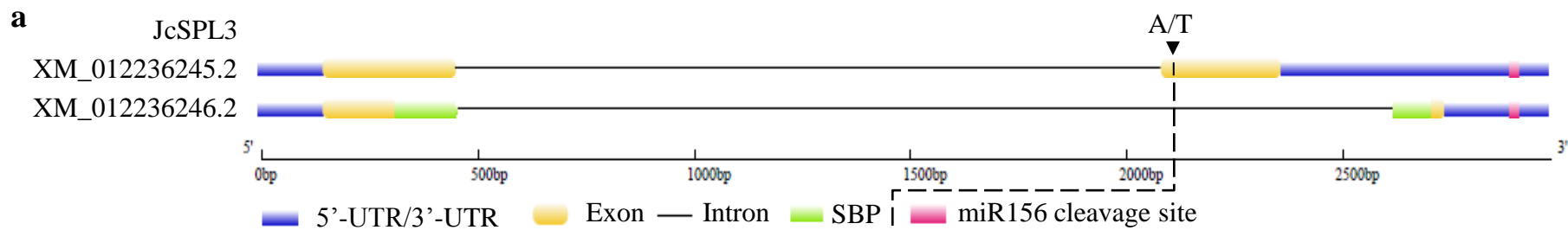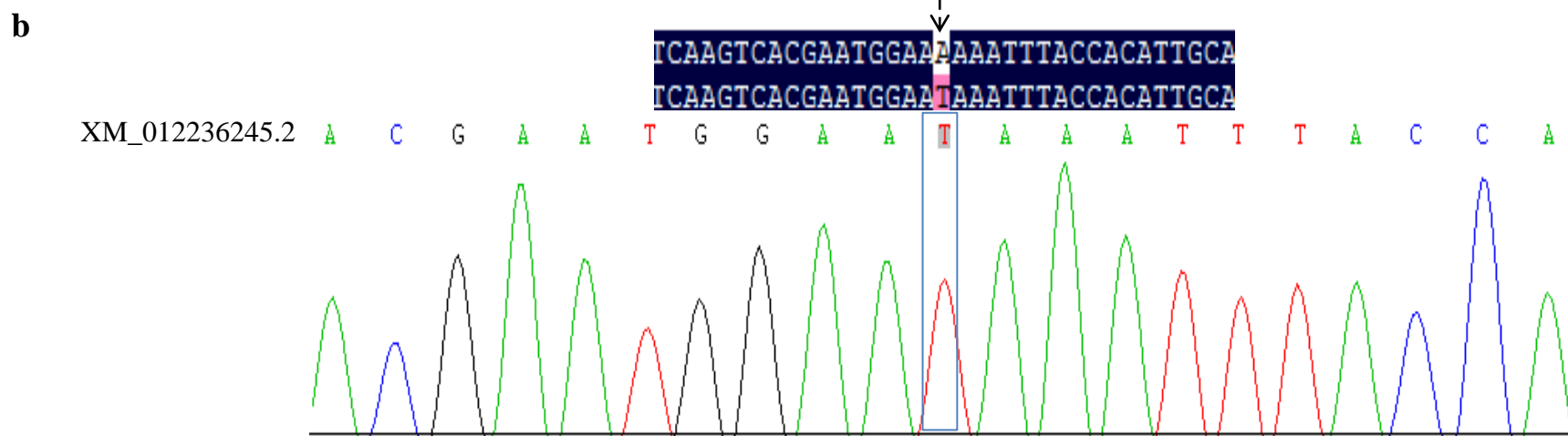

Supplement: Supplementary file 3 — Additional file 3. The predicted gene structure and sequence alignment of JcSPL3. a Schematic representation of the alternative processing of predicted JcSPL3 genes. b Sequence alignment of predicted JcSPL3 and cloned genes (Upper), and chromatogram information. [file 12864_2020_6776_MOESM3_ESM.pdf]

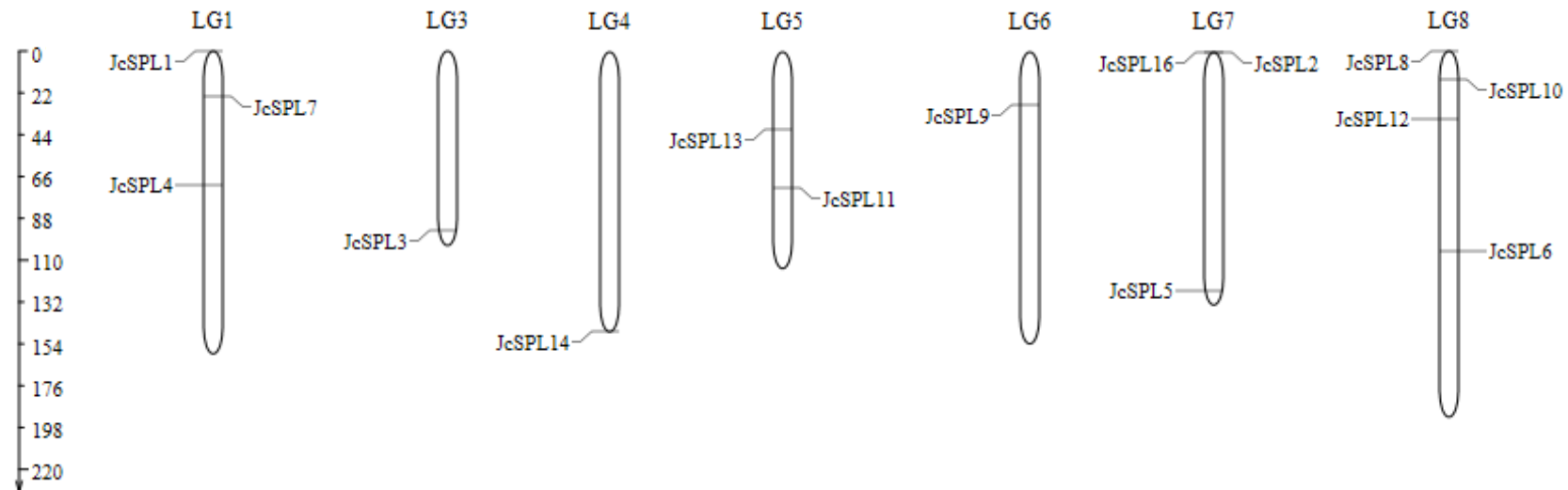

Supplement: Supplementary file 4 — Additional file 4. Chromosomal localization of the JcSPLs. Chromosomal localization of the JcSPLs based on the linkage map. The scale is in centimorgan. [file 12864_2020_6776_MOESM4_ESM.pdf]

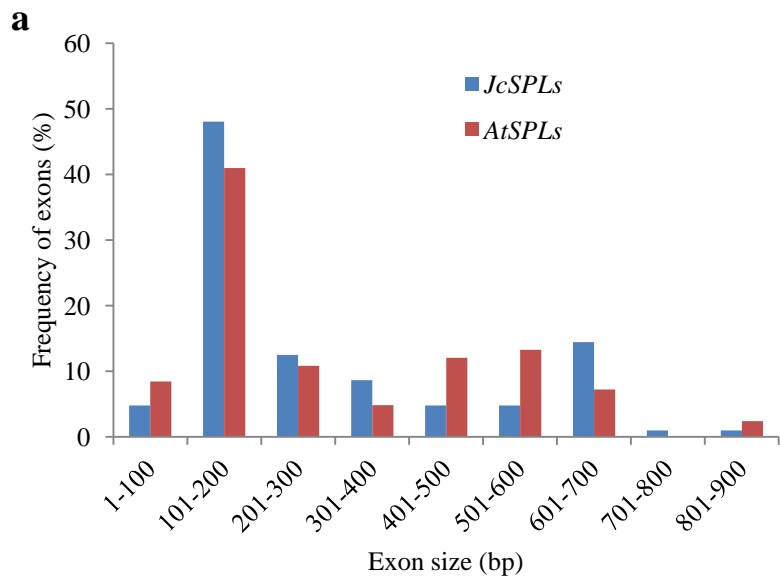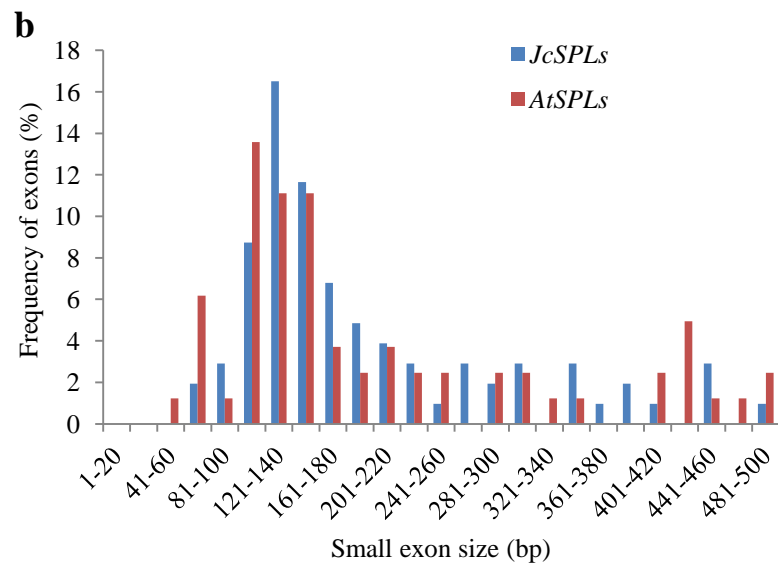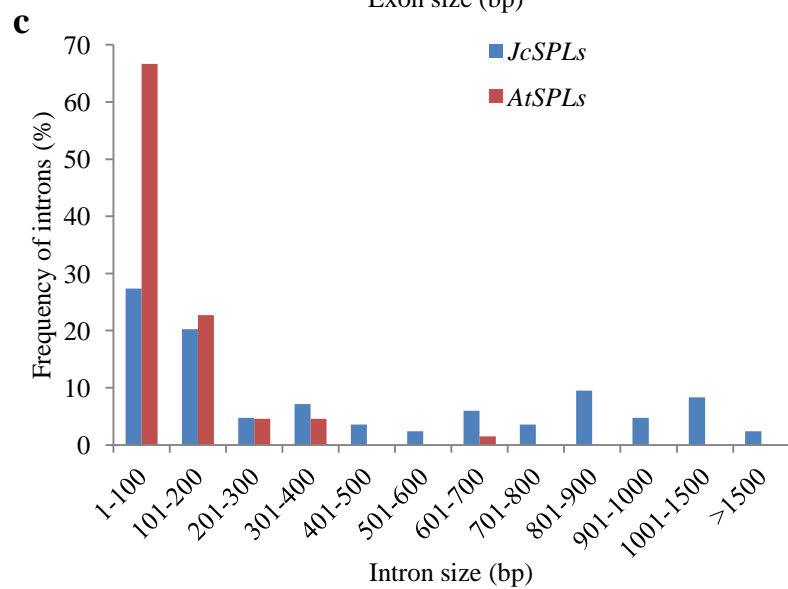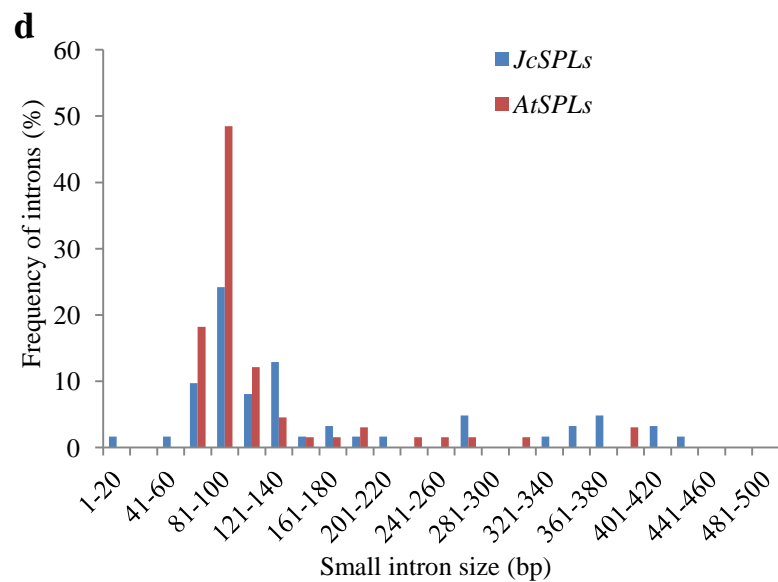

Supplement: Supplementary file 6 — Additional file 6. Size distribution of exons and introns in JcSPLs and AtSPLs. a Size distribution of exons in JcSPLs and AtSPLs. b Detailed size distribution of small exons in JcSPLs and AtSPLs. c Size distribution of introns in JcSPLs and AtSPLs. d Detailed size distribution of small introns in JcSPLs and AtSPLs. [file 12864_2020_6776_MOESM6_ESM.pdf]

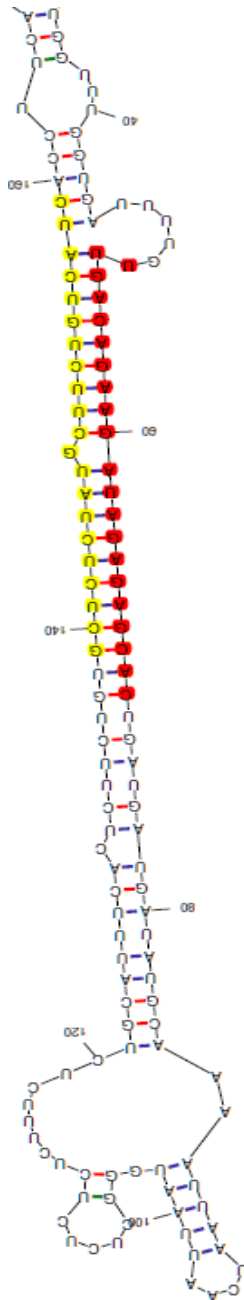

JcMIR156a

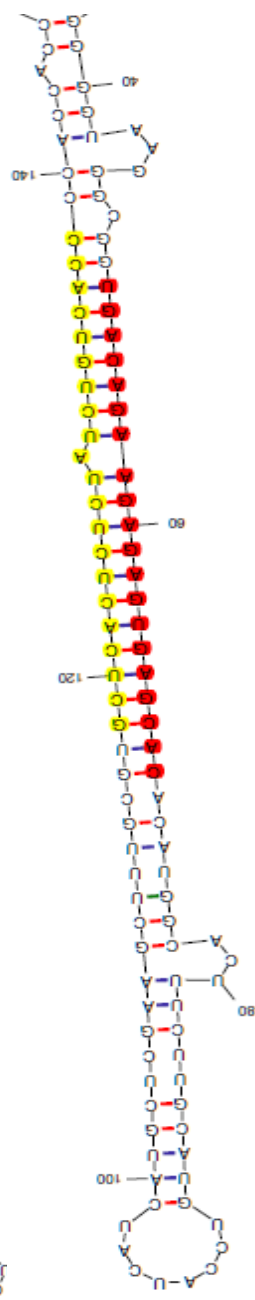

JcMIR156b

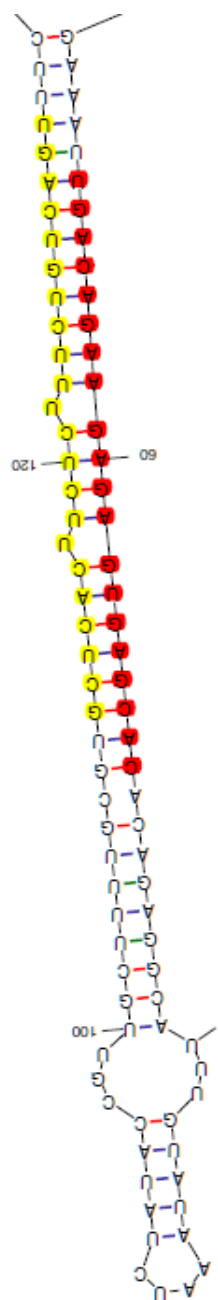

JcMIR156c

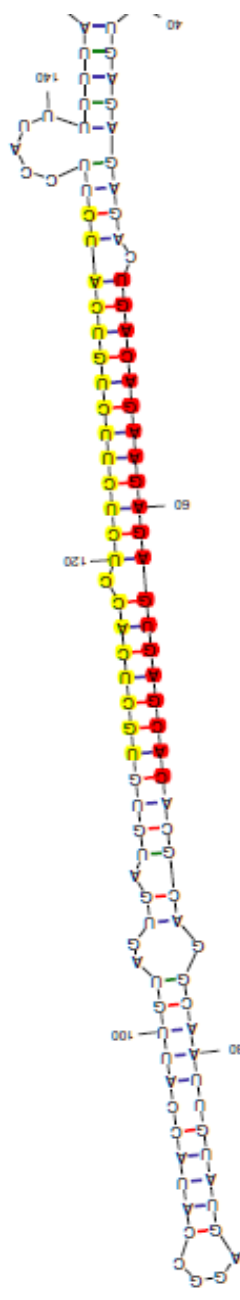

JcMIR156d

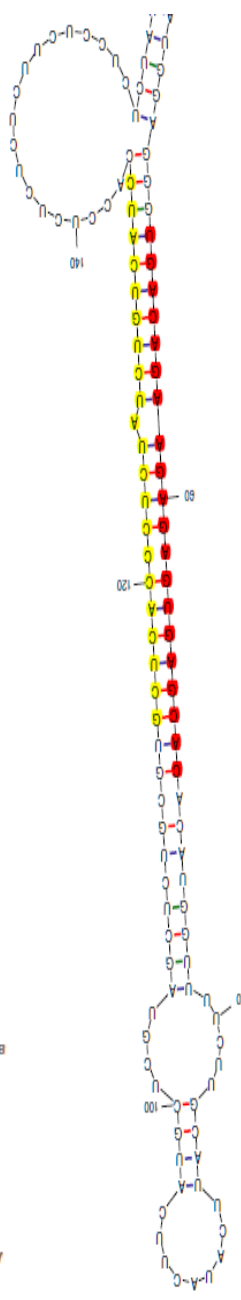

JcMIR156e

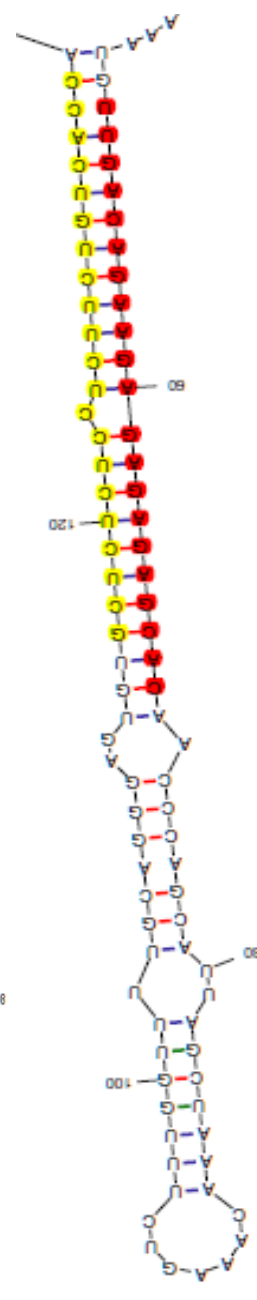

JcMIR156f

Supplement: Supplementary file 9 — Additional file 9. Secondary structure of the JcMIR156 family. [file 12864_2020_6776_MOESM9_ESM.pdf]

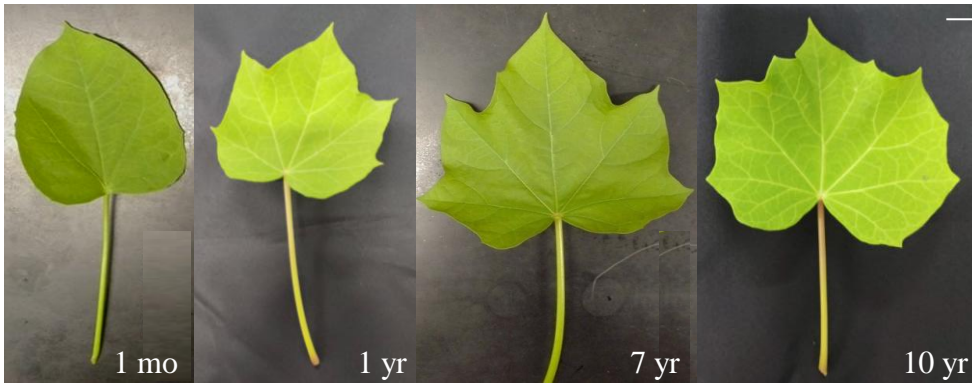

Supplement: Supplementary file 13 — Additional file 13. Leaf morphology in different ages of plants. Scale bars represents 1 cm. [file 12864_2020_6776_MOESM13_ESM.pdf]

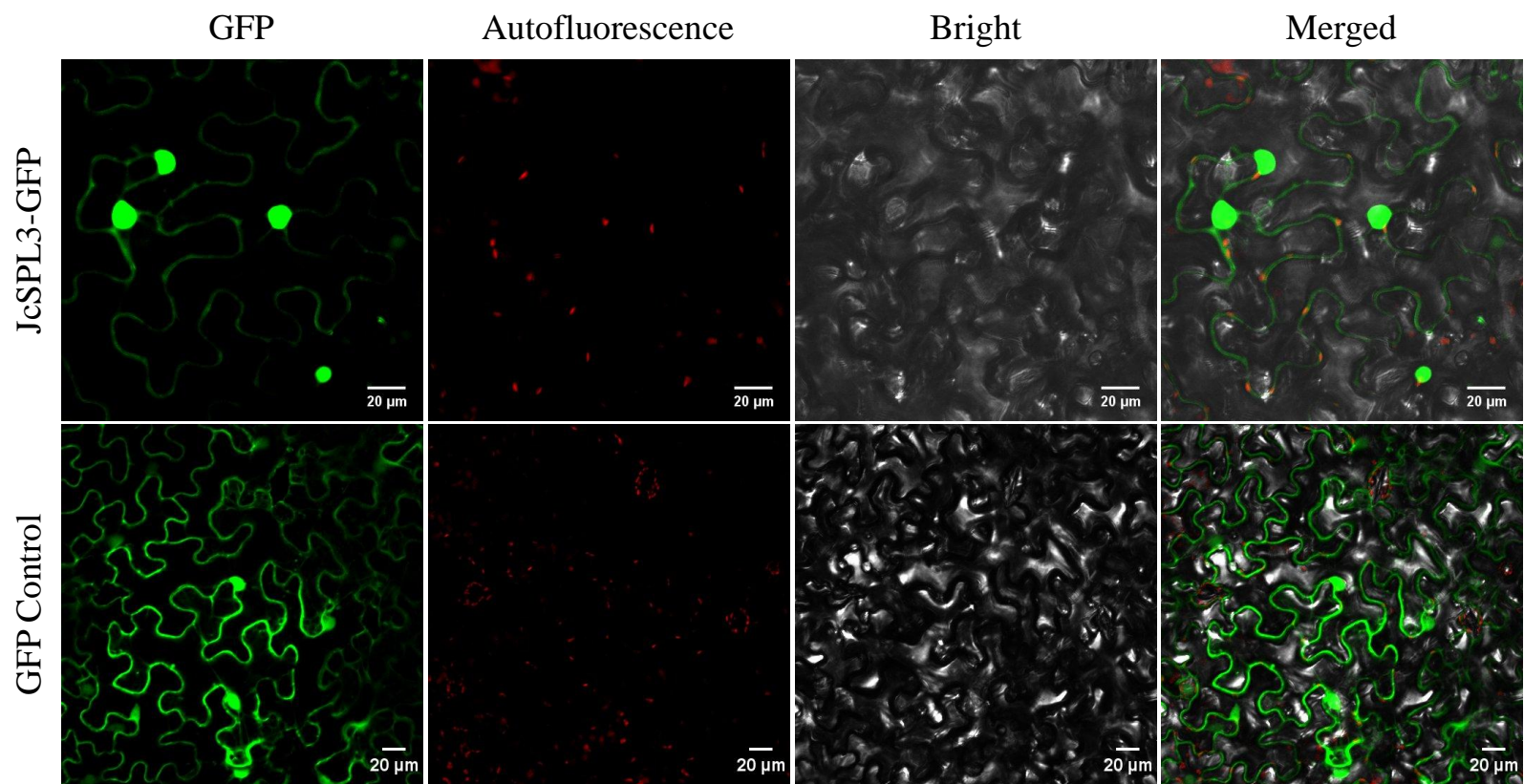

Supplement: Supplementary file 14 — Additional file 14. Subcellular localization of JcSPL3 protein. Confocal laser scanning microscopy of JcSPL3 using GFP-fusion proteins in Nitotiana benthamiana. Merged indicates combined GFP fluorescene and chlorophyll autofluorescene. Scale bars = 20 μm. [file 12864_2020_6776_MOESM14_ESM.pdf]
