# Supplementary material for: Genome-wide characterization of the SPL gene family involved in the age development of Jatropha curcas
Source: BMC Genomics. 2020 May 20;21:368. doi: 10.1186/s12864-020-06776-8 (PMC7238634; doi:10.1186/s12864-020-06776-8)
Supplement: Supplementary file 12 — Additional file 12. Target plots of the targets cleaved by the miR156 family through degradome sequencing. The T-plots show the distribution of the degradome tags along the full-length of the target mRNA sequence (bottom). The red line represents the sliced target trancripts and is marked with arrow. [file 12864_2020_6776_MOESM12_ESM.pdf]

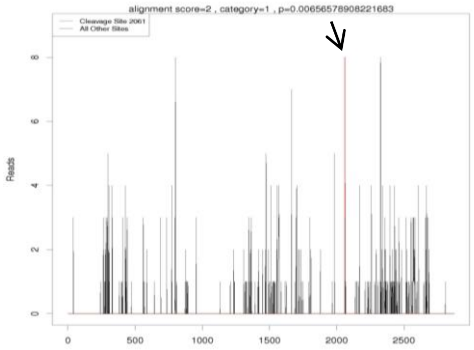

miR156a slicing *JcSPL2* at 2061 bp

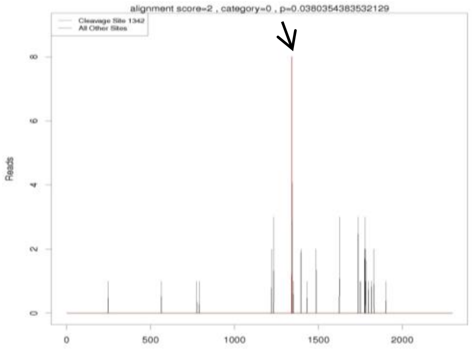

miR156f slicing *JcSPL10* at 1342 bp

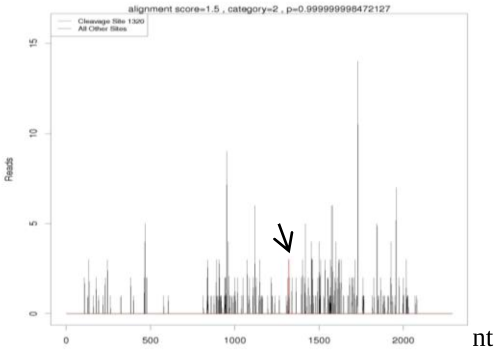

miR156d slicing *JcSPL6* at 1320 bp

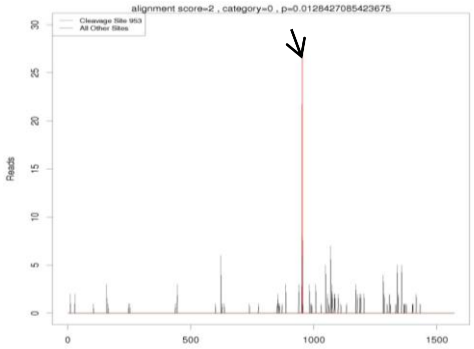

miR156a slicing *JcSPL9* at 953 bp

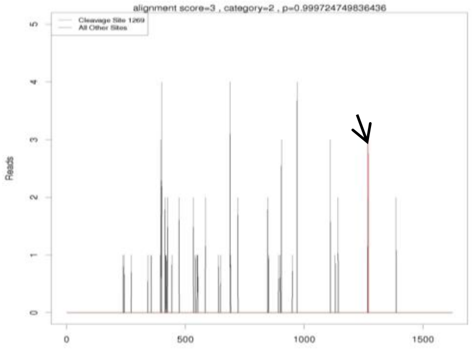

miR156a slicing *JcSPL13* at 1269 bp

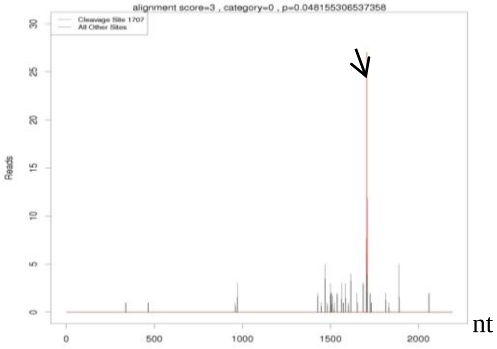

miR156a slicing *JcSPL16* at 1707 bp

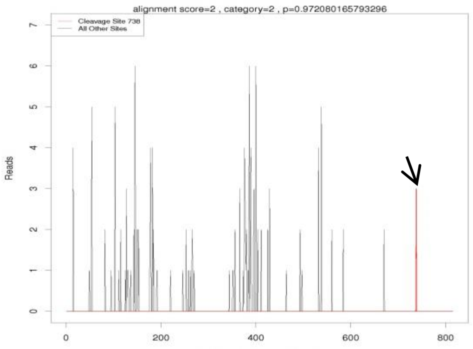

miR156d slicing *JcSPL3* at 738 bp

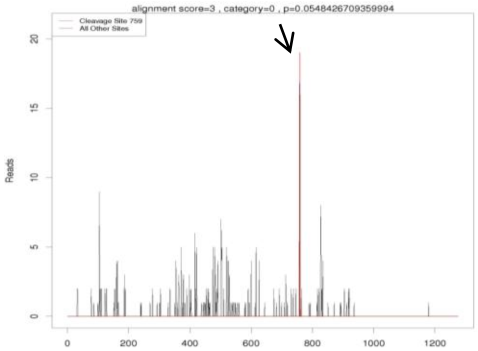

miR156e slicing *JcSPL5* at 759 bp

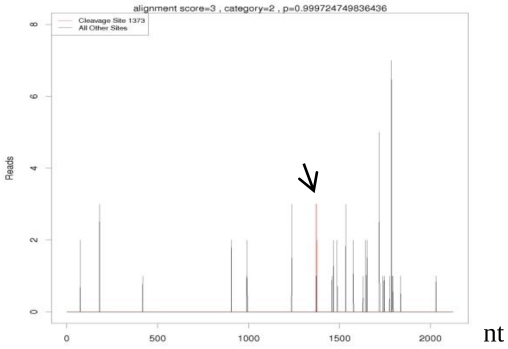

miR156a slicing *JcSPL11* at 1373 bp
